# Science, politics, and health in the brave new world of pharmaceutical carcinogenic risk assessment: Technical progress or cycle of regulatory capture?

**DOI:** 10.1016/j.socscimed.2012.04.043

**Published:** 2012-10

**Authors:** John Abraham, Rachel Ballinger

**Affiliations:** a Department of Sociology, University of Sussex, Brighton, UK; b Brighton & Sussex Medical School, University of Sussex, UK

**Keywords:** Drug testing, Animal models, Pharmaceutical industry, International regulation, Drug safety regulation, Carcinogenic

## Abstract

The carcinogenicity (cancer-inducing potential) of pharmaceuticals is an important risk factor for health when considering whether thousands of patients on drug trials or millions/billions of consumers in the marketplace should be exposed to a new drug. Drawing on fieldwork involving over 50 interviews and documentary research spanning 2002–2010 in Europe and the US, and on regulatory capture theory, this article investigates how the techno-regulatory standards for carcinogenicity testing of pharmaceuticals have altered since 1998. It focuses on the replacement of long-term carcinogenicity tests in rodents (especially mice) with shorter-term tests involving genetically-engineered mice (GEM). Based on evidence regarding financial/organizational control, methodological design, and interpretation of the validation and application of these new GEM tests, it is argued that regulatory agencies permitted the drug industry to shape such validation and application in ways that prioritized commercial interests over the need to protect public health. Boundary-work enabling industry scientists to define some standards of public-health policy facilitated such capture. However, as the scientific credibility of GEM tests as tools to protect public health by screening out carcinogens became inescapably problematic, a regulatory resurgence, impelled by reputational concerns, exercised more control over industry’s construction and use of the tests, The extensive problems with GEM tests as public-health protective regulatory science raises the spectre that alterations to pharmaceutical carcinogenicity-testing standards since the 1990s may have been boundary-work in which the political project of decreasing the chance that companies’ products are defined as carcinogenic has masqueraded as techno-science.

## Introduction

Most social scientists researching pharmaceuticals have devoted attention to clinical trials and post-marketing experiences of medicines, which involve patients/users directly (Abraham & Davis, 2010; Abraham & Sheppard, 1999; Daemmrich, 2004; Epstein, 1996; Fisher, 2009; Healy, 2004; Hedgecoe, 2004; Light, 2010; Pearce, 2007; Petryna, 2009). By contrast, we focus on carcinogenic risk assessment of pharmaceuticals, a branch of animal/cellular toxicology apparently removed from people’s use of medicines, but nevertheless relevant to public health (Tomatis & Huff, 2001). Human exposure to pharmaceuticals can cause cancer, so modern societies have assessed the carcinogenicity of new drugs since the 1960s (Marselos & Vainio, 1991; World Health Organization, 1969). Neither clinical trials nor post-marketing monitoring systems of people’s medicine-use can assess pharmaceuticals’ carcinogenic risks because such risks typically accelerate over the lifespan – 70–90 years for humans – too long for clinical trials, and too late to prevent cancers even if detected by post-marketing monitoring (Schou, 1992, p. 210). Thus, there is considerable need to investigate carcinogenic toxicology beyond clinical trials and patients’ medicine-taking.

Previous social science research on chemical and pharmaceutical risk assessment has examined how techno-scientific standards are applied to particular products by government regulators, and then explained those regulatory interpretations by reference to external socio-political factors (Abraham, 1993, 1998; Brickman, Jasanoff, & Ilgen, 1985; Jasanoff, 1990; Van Zwanenberg & Millstone, 2005). Rather, our focus is on the validation and application of new techno-regulatory testing standards, specifically use of genetically-engineered mouse (GEM) models in pharmaceutical carcinogenic risk assessment. Our research takes this social science field into new empirical domains where regulators must make strategic choices about how much control industry should have over the development of standards.

Of crucial importance is whether the introduction of new GEM models provides a higher standard than before of screening out pharmaceutical carcinogens in the interests of public health or represents a standard that might enable more pharmaceutical carcinogens to reach the marketplace contrary to public health, though in the commercial interests of industry. For decades, regulatory agencies in Europe and the US have been mandated by law to protect public health (Doern & Wilks, 1998; Majone, 1996). We argue that regulatory agencies permitted the drug industry to shape the validation and use of those new GEM tests as screens for pharmaceutical carcinogenicity in ways that prioritized commercial interests over the need to protect public health. Consequently, the limitations of the new tests as public-health protective regulatory science were sustained longer than necessary, until a crisis in their capability to detect carcinogens became extensive, leading to greater regulatory intervention. We contend that this latest episode in the history of pharmaceutical carcinogenic risk assessment can be understood within regulatory capture theory, though to differing degrees in Europe and the US.

In this context, capture theory refers to regulatory agencies’ ‘administrative drift’ towards industry’s commercial interests and away from their mandated regulatory mission to protect public-health interests, together with a cyclical regulatory resurgence when ‘administrative drift’ produces regulatory ‘crises’ – classically a well-publicized drug disaster (Abraham, 1995, 2008; Bernstein, 1955; Carpenter, 2004; Lexchin, 2006). Thus, within capture theory, ‘administrative drift’ (regulatory capture) is not necessarily a permanent state. It might be argued, mistakenly, that deregulatory legislative reforms in the last 15–20 years by EU and US governments have rendered capture theory irrelevant because they have mandated their respective drug regulatory bodies, the European Medicines Agency (EMA) and the Food and Drug Administration (FDA), to facilitate many industry goals.

Certainly, those reforms emphasized regulators’ role in *promoting* health *by approving new drugs* on to the market, as well as *protection* of public health *from* unsafe drugs. The official objective of the EMA, formed in 1995, included ‘to promote public health by providing safe and effective medicines’ (EMA, 1996, p. 9). Meanwhile, a US Congress, committed to increasing pro-business regulation, passed the 1997 FDA Modernization Act, which changed the FDA’s mission statement to include ‘promoting public health by promptly and efficiently reviewing clinical research and taking appropriate action on the marketing of regulated products in a timely manner’ (Carpenter, 2010, p. 731). Increased emphases on faster approvals brought the missions of regulatory agencies closer to industry’s commercial interests, but they did not extinguish regulators’ mandate to prioritize health interests. Both EU and US law continued to *require the EMA and the FDA to protect public health*, while faster drug approvals were *conditioned on promotion of health* (Abraham & Lewis, 2000; FDA Modernization Act, 1997). Thus, the possibility and problem of capture remained even during the deregulatory period of the 1990s and 2000s. Indeed, neo-liberal legislative reforms and capture may have reinforced each other.

An alternative view, often put forward by official representatives of drug regulatory agencies and the pharmaceutical industry, is that the introduction, validation, and use of GEM tests was an example of industry and regulators working and learning together in a scientific quest to improve carcinogenic risk assessment. On this view, the trajectory of regulatory agencies’ action should be understood as that of a ‘learning regulator’ in the face of unfolding scientific developments, rather than in terms of capture theory (Carpenter, 2004). However, we argue that, in this context, the ‘learning regulator’ representation was part of science-politics ‘boundary-work’, which facilitated regulatory capture of carcinogenic risk assessment by enabling industry scientists to define commercial concerns as matters of techno-scientific progress, and to shape some standards of public-health policy according their institutional priorities (Jasanoff, 1990). Subsequently, the consequent industrial science struggled to meet the task of public interest regulation, so such boundary-work became less feasible as the worrying implications for public health of GEM tests’ use in drug development became more compelling among the wider scientific and regulatory communities. Consistent with capture theory, we suggest that the regulatory resurgence, which followed, exhibited reduced concern to accommodate industry interests and was an attempt by regulators to reassert their reputation as guardians of a regulatory science intended to screen out carcinogenic dangers to public health, rather than solely a result of learning more about the science. To examine the interest-politics of the introduction of GEM tests into drug development, and the applicability of capture theory therein, we investigated the financial/organizational control, methodological design, and interpretation of results, of the GEM tests’ validation process; and considered the types of GEM tests selected for use by industry, the issues that attended industry use, and the responses by regulators and experts to the outcomes of such use.

## Background

The idea of incorporating GEM models into pharmaceutical carcinogenicity-testing standards was established at the International Conference on Harmonisation of Technical Requirements for Registration of Pharmaceuticals for Human Use (ICH) during the 1990s (Abraham & Reed, 2003). Formed in 1990, ICH is an organization/network consisting of expert scientists representing the pharmaceutical industry associations and government regulatory agencies of the EU, US and Japan (Abraham, 2009). According to its secretariat, the International Federation of Pharmaceutical Manufacturers’ Association (IFPMA), ICH aimed to ‘harmonize’ different techno-regulatory drug testing requirements across the three regions to increase efficiency in drug development and regulation by eliminating unnecessary duplication in testing without compromising drug safety – a claim uncritically accepted by some scholars (Daemmrich, 2004; pp. 157–160; Vogel, 1998). However, regarding carcinogenicity testing, ICH evidently sought to *reduce*, not ‘*harmonize*’, standards because by the late 1970s, regulatory agencies in North America, Western Europe, and Japan already all had the same standards (Abraham, 1998).

Industry participants and regulators from Europe and Japan at ICH aimed to decrease the number of long-term rodent carcinogenicity tests required before marketing approval from two species (rats and mice) to just one species, the rat (Abraham & Reed, 2003). Typically, lasting 18–24 months, the long-term rodent tests sought to examine drugs’ carcinogenic effects over most of the lifespan of the test animals. They were the most expensive and time-consuming aspect of drug testing not involving patients in trials or epidemiological studies. For decades they were central to screening for *non-genotoxic* pharmaceutical carcinogens, which may initiate and promote tumour formation, but do not cause the mutations thought to initiate tumour formation. The long-term studies were particularly important health screens because *non*-genotoxic carcinogens are not detected by the inexpensive battery of quick *in vitro* mutagenicity tests on micro-organisms and disembodied human cells used to identify *genotoxic* carcinogens that cause cancer primarily by damaging DNA.

Initially at ICH, the pharmaceutical industry and European regulators proposed that the long-term carcinogenicity test with mice should be jettisoned by claiming that mouse tumour findings were not relevant to human risk or regulatory decisions about new drugs (Emmerson, 1992; Usui, Griffiths, & Lumley, 1996; Van Oosterhout et al., 1997). (**1**) However, the FDA rejected that claim and the proposal to conduct carcinogenicity testing in only one rodent species, arguing that such tests were required in more than one species to identify trans-species carcinogens – the ones posing greatest threat to humans (Contrera, Jacobs, & DeGeorge, 1997). The FDA’s unwillingness to accept carcinogenicity testing in just one rodent species indicates that it was significantly less captured than EU regulators at ICH. Nonetheless, the FDA agreed to a compromise with industry and Europe, which involved replacing the long-term mouse test with new GEM tests lasting just six months, even though ICH experts acknowledged that the new tests had never been validated for carcinogenicity screening (DeGeorge, 1996; MacDonald, 1998; Mitsumori, 1998). In 1997, ICH approved a new techno-regulatory standard, which permitted pharmaceutical firms seeking marketing approval to conduct only one long-term carcinogenicity test in rats, provided that it was accompanied by an appropriate alternative short-term GEM test (Abraham & Reed, 2003). Arguably, industry had used ICH as a vehicle for partial capture of the FDA.

Here we provide the first social scientific investigation of how these new GEM tests have subsequently been used by the pharmaceutical industry and regulators. Abraham and Reed (2003) analyzed debates about carcinogenic risk assessment together with the emergence of the idea of GEM tests at ICH. Drawing on separate research, we report fresh findings addressing the period after 1998, involving the activities of the International Life Sciences Institute (ILSI) and the Alternative to Carcinogenicity Testing (ACT) programme during the 2000s. We surpass Abraham and Reed’s (2003) analysis of the construction of the GEM testing standard to examine the socio-political and health significance of its validation and actual use.

## Data sources and research methods

The research spanned 2002–2010, including years of fieldwork in the EU and US. It met ethical requirements of the Wellcome Trust, who funded it. The research did not require formal institutional ethical approval because it did not involve patients or data directly relevant to patients. The two main methods were literature review and in-depth interviews. For example, PubMed was searched electronically (1965–2010) using keywords, ‘carcinogenicity testing’, ‘risk assessment’, ‘transgenic animals’, and ‘genetically altered mouse models’; while *Scrip* (1998–2010) – the twice-weekly pharmaceutical trade newsletter – was searched manually. Email notifications of latest developments extended the literature review to ongoing organizational practices via consultation documents/drug labels/letters/notices of meetings/newsletters/press releases. Documents produced by relevant organizations (drug regulatory agencies, expert advisory committees, pharmaceutical companies/associations, government research institutes, universities, cancer organizations, patient associations, professional toxicology societies, and contract services) were also analyzed.

Interviews were conducted in Europe and the US. The number of UK interviews was relatively large because the EMA is London-based, the UK has one of the largest pharmaceutical industries in Europe, and the research-team was UK-based. In addition to geographical location, interviewees were selected on primary criteria as ‘informants’ and on secondary criteria as ‘respondents’. Primary criteria referred to knowledge/expertise derived from involvement in ICH and/or subsequent validation/use of the new testing regime and/or authorship of publications about carcinogenicity testing. Secondary criteria related to institutional affiliation, such as ‘pharmaceutical industry’; ‘government regulators’, ‘expert research scientists’ working in universities/government institutes, and ‘other interest groups’, such as patient/consumer organizations. Interviewees with multiple affiliations were allocated a ‘primacy’ categorization most relevant to our study.

Ninety-three interview requests yielded 53 interviews (57% response rate), 22 received no reply, and 18 were declined. Table 1 shows the response rates per region, by primacy categorization of interviewees. Informed consent was gained by providing interviewees with an outline of the study, the issues to be covered in the interview, and the likely use of both. Lasting 1–2 h, interviews were semi-structured to allow impromptu follow-up inquiries, as well as pre-planned core questions, which formed the interview protocol, including topics such as ‘interviewee biography’, ‘animal lifespan studies’, ‘ICH’, ‘GEM tests’, ‘ILSI’, and ‘GEM tests’ evaluation-in-use. All were tape-recorded and transcribed, except for three when contemporaneous field-notes were employed. Data were analyzed independently by the authors with assistance of a coding frame and Atlas computer software, designed for qualitative data management. Codes included ‘ACT organization’, ‘ACT study and outcomes’, ‘GEM use’, ‘GEM evaluation’, ‘regulatory–industry interaction’, and ‘validation’.

## Regulatory retreat and industrial capture of validation

The new short-term carcinogenicity tests involved mice, which were genetically-manipulated by either introduction of genes associated with human cancer (known as oncogenes), or removal (‘knocking out’) of genes thought to suppress tumour development, known as tumour-suppressor genes (Tennant, 1996). Scientists’ understanding of mechanistic routes of human carcinogenesis informed the types of genetic manipulation involved (**2, see Interview Notes box**). The techno-scientific hypothesis underpinning the GEM tests was that tumour ‘initiation’ was built into genetically-engineered mice enabling carcinogenic effects to be detected much sooner than in ‘normal’ rodents because only later stages of carcinogenesis needed to occur during the experimental period of drug testing. On this hypothesis, a carcinogenic drug should be detected quickly by GEM tests because ‘initiated’ animals should develop more cancer tumours more rapidly than ‘normal’ mice (Schou, 1992). ICH outlined three main types of GEM tests: the tgAC model involving transgenic mice with the oncogene, v-Ha-ras, introduced; the rasH2 model using transgenic mice with the oncogene, cHa-ras; and the p53 model involving ‘knock-out’ mice with the tumour-suppressor gene, p53, removed.

When ICH approved GEM tests into pharmaceutical regulation in 1998, the tests’ capability to accurately identify carcinogens had never been validated. This drew criticism from regulators and academic scientists outside ICH, who regarded inclusion of un-validated tests in regulatory guidance as premature (**3**) because it ‘lacked appropriate scientific rigour’ and was ‘bad science’ (**4**) (UK Department of Health, 1997, p. 113). Even some ICH experts acknowledged that validation was needed (MacDonald, 1998, p. 272). Consequently, during the early 2000s, validation studies were conducted mainly in the US. (**5**) In considering whether the validation process was captured by industry interests we examine evidence regarding how the process was controlled, designed, and interpreted.

### Organizational ‘ownership’ and control of expertise

The American, European and Japanese regulatory agencies pledged publicly that ICH’s harmonization would not compromise drug safety. In making that pledge, they acknowledged that test standards for pharmaceuticals were matters of public health for which regulatory agencies continued to have central responsibility mandated in law by their legislatures. All the more so regarding carcinogenicity testing of pharmaceuticals for non-life-threatening conditions because the health-promoting effects of rapid drug approval could scarcely outweigh serious risks of cancer. Yet the regulatory agencies permitted the pharmaceutical industry to control the process with which the new GEM tests were ‘validated’. While the pharmaceutical industry has long controlled the routine testing of individual drug products in long-term rodent studies for marketing purposes, efforts to validate the predictive value of the long-term rodent model *as a test standard*, have frequently involved government agencies, most notably the US National Cancer Institute and National Toxicology Programme, and the Europe-based International Agency for Research on Cancer (Haseman & Huff, 1987; Huff, Jacobson, & Lee Davis, 2008; Marselos & Vainio, 1991). Hence, the extent of industry control of the ‘validation’ of GEM tests is a reasonable indicator of capture.

The validation process became known as the ‘Alternative to Carcinogenicity Testing (ACT)’ programme, whose core funding derived from thirty companies, mainly in the pharmaceutical sector, (**6**) involving 55 laboratories at a cost of US$35 million, (**7**) under the auspices of the industry-funded International Life Sciences Institute (ILSI) in the US (Robinson & MacDonald, 2001, pp. 3–4). While some of ACT’s subcommittees included advisers from academia, government research, and regulatory agencies, all nine members of its formal Steering Committee were from industry, including leading ICH figures, who had argued for the elimination of long-term carcinogenicity tests in mice (Cohen, Robinson, & MacDonald, 2001; Robinson & MacDonald, 2001).

Such industry dominance shaped the expertise drawn upon to participate in the validation process. The WHO’s International Agency for Cancer Research (IARC), an independent body, was not consulted, despite longstanding and internationally-recognized expertise in carcinogen identification and categorization. A leading industry proponent of ACT justified IARC’s exclusion on the grounds that he disagreed with IARC’s categorization of particular chemicals as human carcinogens, characterizing IARC as ‘political’, rather than scientific. (**8**) Thus, some scientists managing the ACT validation process were reluctant to involve experts perceived to be more likely than themselves to interpret pharmaceuticals as posing carcinogenic risk. The consequence was to justify this industry agenda by shifting the science-politics boundary so that such experts would fall outside an ostensibly ‘legitimate techno-scientific’ agenda.

### Shaping of methodology

Industry’s primary concern informing the methodological design was that GEM tests might produce results propelling scientists to designate even more pharmaceuticals as presenting carcinogenic risk than the long-term mouse studies had done. (**9**) That scenario could have posed serious commercial drawbacks offsetting any gains derived from the GEM tests’ lower costs and shorter duration. According to scientists across the sector, ‘the ILSI [ACT] study was an attempt by the industry to convince themselves that these [GEM] assays were not overly sensitive’, that is, they would not produce more results that industry scientists believed were false positives by ‘mis-identifying’ (as industry saw it) non-carcinogens as carcinogens. (**10**)

The pharmaceutical industry’s agenda to check that the new GEM tests did not produce more false positives than the long-term rodent tests explains why most of the compounds selected for the validation process were non-carcinogens. (**11**) Of the 21 compounds selected, only six were human carcinogens (defined according to established genotoxicity tests, human epidemiological findings and long-term animal studies), while 15 were non-carcinogens in humans (Robinson & MacDonald, 2001). Yet when screening for carcinogens to protect public health, the priority is to have test systems able to detect human carcinogens, and not miss them (false negatives), so that regulatory agencies can limit/prevent human exposure to the drug. However, the ACT programme invested more than twice as much effort (15:6) in checking the validity of GEM tests according to industry’s commercial interests (not too many false positives regarding non-carcinogens) relative to the interests of public-health protection (not too many false negatives regarding human carcinogens). That this selection reflected industry bias in the validation process was confirmed by a former FDA senior scientist, who noted that regulators would have selected different compounds if they had managed the process, but ‘the FDA went along with it [the ACT selection]’ to accommodate industry (**12**) – further indication of a capture trajectory showing that the regulators were aware of the scientifically problematic nature of the validation process, rather than merely on a learning curve.

### Interpretation of results

Some of the 21 compounds in the ACT programme were tested in more than one GEM model, so the number of tests exceeded 21. Across the six known human carcinogens, involving 32 tests, GEM models correctly identified these carcinogens in only 17 (53 per cent), produced nine false negatives (28 per cent), and 6 (19 per cent) equivocal results. Hence, in nearly half the cases, GEM tests failed to identify human carcinogens – the regulatory capability needed to screen pharmaceuticals for public-health protection. By contrast, they correctly identified 82 per cent of compounds that were both human *non*-carcinogens and rodent carcinogens, and 100 per cent of compounds that were *non*-carcinogens in both humans and rodents (Eastin et al., 2001; Storer et al., 2001; Usui et al., 2001; Van Kreijl et al., 2001).

Many in the pharmaceutical industry and government regulatory agencies involved with ACT found the results reassuring (Goodman, 2001, p. 174). (**13**) Apparently a method of carcinogenic risk assessment had been found that would lessen workload for regulators and result in faster and cheaper drug development for industry with no greater commercial risk than before of denial of marketing approval by regulators because of positive carcinogen identification, false or otherwise. The validation process implied that GEM tests could be aligned with industry’s commercial interests and some institutional interests of regulators. However, as many toxicologists in the US acknowledged, evidence that GEM tests offered any scientific improvement over, or could even adequately replace, the long-term studies in mice to screen for potential non-genotoxic human carcinogens in the protection of public health was, at best, scant, especially given a mere 53 per cent detection rate (Cohen, 2001, p. 188; Cohen et al., 2001, p. 18; Pettit, 2001). (**14**) Even the US National Institute of Environmental Health Sciences (NIEHS), where many GEM tests were developed, found that GEM tests missed significantly more known/probable human carcinogens than the two-rodent-species lifespan studies, though a GEM test plus a long-term carcinogenicity study in rats fared better (Pritchard, French, Davis, & Haseman, 2003). Moreover, after the ACT programme’s completion, the UK expert Committee on Carcinogenicity (UKCoC) concluded that none of the GEM models were suitable to replace long-term mouse studies (UKCoC, 2003). (**15**) Yet, the EMA and the FDA, accepted ACT’s favourable interpretation of the ‘validation’ results and permitted industry to continue to control the nature of the GEM tests in use for drug development. We contend that this reflected capture, rather than solely a trajectory of technical learning because good scientific reasons had already emerged to suggest regulatory caution about GEM tests’ capabilities to protect public health.

## Using the mouse models: industrial science eclipses public health

By 2003, the FDA reported that a quarter of the proposed mouse carcinogenicity studies it received from companies were GEM tests instead of a long-term mouse study (MacDonald et al., 2004). Twenty-four completed GEM tests (p53, tgAC, and rasH2) were reviewed by the FDA between 1998 and 2003, though only eight by the EMA (Jacobson-Kram, Sistare, & Jacobs, 2004; Van der Laan, 2003). About three-fifths of these were p53 and a fifth were tgAC. Less frequent use of the tgAC test may be because regulators stipulated that it was suitable to assess only carcinogenicity of *dermal* pharmaceutical products (Jacobson-Kram et al., 2004, p. 51; MacDonald et al., 2004). Use of the Japanese rasH2 model was sparse in Europe and the US (MacDonald et al., 2004).

The regulatory agencies’ representation of GEM-tests’ introduction as merely a scientific quest to improve carcinogenic risk assessment was consistent with their mandate to protect public health. However, examination of industry’s preoccupations when using the tests reveals that that representation is better understood as boundary-work, which rendered commercial priorities indistinguishable from regulatory science and assisted the continued industry capture of carcinogenic risk assessment. As we show in the remainder of this section, in industry hands, the costs of the new science and its potential to enhance product success drove the nature and extent of GEM tests’ use, rather than optimal advances in public-health protection.

### Costs and savings

Despite the NIEHS’s finding that without long-term rat studies, GEM tests failed to detect a considerable number of human carcinogens, the Chair of the ACT programme’s Steering Committee, a senior industry toxicologist, proposed that the ‘core battery’ of carcinogenicity testing should include *in vitro* mutagenicity tests combined with GEM models, but exclude any long-term rodent studies at all – which he relegated to ‘ancillary studies … not required unless all other data are inconclusive’ (MacDonald, 2004). That proposal seemed to encapsulate a widespread industry view that relying on only one long-term study instead of two was merely a stepping-stone towards elimination of all long-term rodent studies – a direction of travel aided by the shift in ‘conceptual power’ established by the ICH compromise (Carpenter & Tobbell, 2011).

The stepping-stone was intended to reduce the costs and duration of drug development. Although GEMs were ten times more expensive than ‘normal’ experimental mice, the smaller number of animals requiring fewer feeding, housing, and labour/pathology costs than the long-term mouse study was estimated to deliver a 30–50% saving – approximately US$750,000 per drug. (**16**) More importantly, time saved by the shorter GEM tests potentially allowed a new block-buster drug an additional year or more of post-market patent protection from generic competitors that could be worth a billion dollars to the manufacturer. (**17**)

### Product security

Neither the small size nor short duration of GEM tests was the crucial element of commercial interest for pharmaceutical companies. One industry scientist elaborated that such savings ‘may not be nearly as important as getting the type of results you want, and getting them when you want, to the satisfaction of regulatory agencies’. (**18**) Confidence that a carcinogenicity test was not going to jeopardise marketing approval of their new drugs was the most important consideration for companies because profits from marketing had the potential to dwarf the development costs of testing, especially non-clinical testing. (**14**) That explains why the industry invested heavily in a validation process designed to check that GEM tests would not generate an excessive number of ‘false’ positives.

Pharmaceutical firms’ emphasis on obtaining favourable outcomes affected their choice of carcinogenicity test, made carefully to avoid ‘false’ positives because ‘you might turn up results that you needn’t have produced [with a different test]’. (**19**) As one senior industry scientist involved with ACT disclosed, pharmaceutical companies feared positive results in GEM tests more than in long-term mouse studies because their scientists had become adept at challenging the significance of some positive tumour findings in long-term mouse tests to human carcinogenic risk:

What do you do with the positive? That’s what really terrifies much of the pharmaceutical industry. If I get a positive finding in the two-year [long-term] mouse bioassay, I understand what to do with it. … If I get a positive result on a p53 mouse – the emotional response to that by the regulatory community is much greater… than a positive [long-term] mouse bioassay. In today’s world, a positive finding in the livers of mice after two years [long-term study] is of virtually no regulatory consequence – it’s written off because we’ve taken three decades to understand it and get comfortable with it. (**8**)

Thus, avoidance of positive results in GEM tests was particularly important to the industry because it had not developed sufficient explanations to attribute GEM tumours to some cause other than the test drug. It may be noted that the FDA, unlike European regulators, did not accept that liver tumour findings from long-term mouse studies were irrelevant to human carcinogenic risk. Nonetheless, a positive result in GEM tests was likely to be *more* damning for a drug’s prospects than liver tumours in mice over a long-term study.

### Flexibility in test scenarios

Many in industry favoured GEM tests over long-term studies because of increased flexibility over drug testing. For example, because a short-term GEM test could be started later in the drug development process than a long-term rodent study, if early clinical trials indicated that the drug should be abandoned, then the company could avoid any *in vivo* carcinogenicity testing. (**18**) Alternatively, GEM tests could be used early in drug development before clinical trials, as pre-screening for a long-term study, (**8**) or used later to alleviate concerns about the adequacy/results of a long-term study in rats (MacDonald et al., 2004).

Further indication that the EMA was more captured than the FDA regarding these matters is the finding that industry scientists and some EU regulators expressed concern that the FDA might require GEM tests to be conducted before the later, large-scale clinical trials, known as phase-three trials. That scenario would limit a firm’s flexibility and could delay the drug development process. (**1**) Yet, as FDA scientists pointed out, completion of GEM tests before phase-three clinical trials (sometimes lasting a year or more) could prevent exposure of many patients to potential carcinogens (Jacobs & Jacobson-Kram, 2004). (**20**) Evidently, the FDA was not completely captured and by 2004 had become more interventionist about GEM tests. However, many other aspects of industry use of GEM tests remained unregulated.

For example, industry scientists strongly opposed the use of GEM tests as a pre-screening regulatory requirement before beginning a long-term study in rats because that scenario would deny firms flexibility in interpreting results across different carcinogenicity tests. (**21**) Furthermore, such pre-screening would delay drug development by over six months, with adverse commercial implications. (**7**) Consequently, drug firms’ commercial interests propelled them to use GEM tests concurrently with a long-term rat study, which could be terminated after six months if GEM tests were positive. (**12**) Concurrent screening generated inferior knowledge-acquisition that was less likely to maximize public-health protection because the long-term rat study could not be designed according to what was learned from the GEM test. (**22**) Nonetheless, such practices remained unchallenged by regulators.

## Regulatory resurgence and reputational control: the unravelling of industry science

For 60% of pharmaceuticals subjected to the tgAC test during their development, and submitted to the FDA, the test produced positive results, often while long-term rat studies were negative. Hence, the test could frequently undermine new drug development, which may account for its unpopularity with industry. (**23**) As tgAC test results derived from the skin, alternative causes of positive carcinogenicity findings were put forward, such as tumour development due to mice’s grooming and wounding, purportedly making the test ‘too sensitive’ for risk assessment. (**24**) Consequently, this GEM model became increasingly marginalized in pharmaceutical development and regulation, not least because industry rarely selected it for use. (**8**,**11**)

With tgAC’s marginalization, the p53 test became of paramount importance and by far the most widely used in drug development. Use of the p53 test drew few complaints from companies. It was rarely positive, so did not usually threaten the viability of pharmaceutical products. (**17**,**25**) However, if those many negative p53 test results included false negatives, then that could imply public exposure to undetected carcinogens. As we show in this section, regulators responded to that problem with increased oversight of the design and use of GEM tests in drug development, but only after it had reached crisis proportions. Given the techno-scientific limitations of GEM tests to screen for human carcinogens, even at ‘validation’, such regulatory intervention might have been expected earlier if the regulatory agencies were merely on a learning curve about how to improve carcinogenicity testing in the interests of public health, rather than engaged in a regulatory resurgence to re-establish their reputation.

### Regulators confront dysfunctional industry science

Specifically, by the mid-2000s, for all five of the new drug applications received by the EMA, and in all but one of the 17 received by the FDA, using the p53 test, the result was negative, which then contributed to an overall drug evaluation alongside long-term rat studies (MacDonald et al., 2004). Although the one positive p53 result led to withdrawal of the drug from the market, evidently the p53 test was almost always used to confirm a negative, or question, a positive long-term carcinogenicity study in rats. (**26**) For example, p53 tests were negative for drugs found to generate lymphomas, kidney tumours and/or testicular neoplasms in long-term rat studies (Sistare & Jacobs, 2003). Moreover, most of the 16 drugs submitted to the FDA with negative p53 results were found to be positive in the standard battery of *in vitro* genotoxicity tests, as well as positive in long-term rat studies. Even the staunchest supporters of the p53 model in government agencies and the industry could not render those kinds of negative results as credible for regulatory decisions. As one expert regulatory advisor noted, actual use of the p53 test suggests that it may be producing ‘lots of false negatives’. (**27**) Similarly, an FDA regulator remarked: ‘what we’re finding out now is that very few things are positive in it [p53] – so now regulators are saying, “it’s not sensitive enough”’. (**17**) To maintain an adequate reputation for public-health protection, regulators began to press an agenda for longer and larger GEM tests thought less likely to miss carcinogens.

### Streamlining in reverse – a regulatory science for public health

Regulators’ growing concerns about false negative results with p53 amounted to a scientific crisis about the test’s validity. Questions were asked about whether the test-model was too short-term, involved too few animals or was too mechanism-specific for regulatory screening. (**28**) While the short-term duration of p53 and other GEM tests had commercial advantages for pharmaceutical firms, it also meant that exposure during chronic ageing was avoided, possibly overlooking drug carcinogenicity manifest during the ageing process. (**12**) Hence, increasingly scientists proposed that p53 tests should be extended from six to nine months duration in the hope of improving their detective capability.

Reductions in animal numbers from 50/sex/dose-group in lifespan studies to just 15 in GEM tests was a promising financial gain for industry, though ‘not necessarily an improvement in the science’. (**29**) Nevertheless, partly in response to the prevalence of p53 negatives, from the mid-2000s, the EMA and FDA recommended an *increase* in the size of GEM tests from 15 to 25 animals/sex/dose-group to improve the tests’ ‘statistical power’ to register significant carcinogenic effects. (**11**,**18**) Such changes, however, eroded some of the ostensible advantages of GEM tests over long-term studies, including savings for industry. As relentlessly negative results from p53 tests became increasingly non-credible for protecting patients from carcinogens, regulators were no longer willing to stake their reputation on that industrial science.

### GEM tests as regulatory science in crisis

Most significantly, the problem of extensive false negatives with p53 tests generated fundamental crises of confidence in the mechanistically-oriented, GEM model approach to regulatory screening for pharmaceutical carcinogens. As one industry scientist succinctly put it:

At most, you’ve got three mechanisms of carcinogenesis that are represented in these [GEM] models. That’s not the only way cancer can be produced. (**5**)

Indeed, in 2000, the US National Cancer Institute listed over 4500 different genes related to cancer. One FDA regulator explained why this scientific crisis threatened public health and the viability of GEM testing for industry:

Few chemicals are carcinogenic by only one mechanism. So if you tested it [drug] in two or three transgenic models, it could still be carcinogenic even though it was negative in all of them. That’s the problem and we do not have enough transgenic models that are useful and validated to line up against all potential mechanisms of carcinogenesis, and even if we did, what good would that be? Because when you have an unknown, are you going to test them [new drugs] in 20 different transgenic models? No, … you would stop using transgenic models, and just go back to the two-year [long-term studies].(**2**)

The hypothetical scenario of using 20 different GEM tests for each drug development would also be more costly and time-consuming than a long-term study. (**30**)

The extent of false negatives and positives resulting from GEM tests led some scientists to conclude that the problem of identifying non-genotoxic carcinogens relevant to human risk ‘still exists [because] these [GEM] models have not proved good discriminators between many non-genotoxic and genotoxic compounds’, (**3**) and have not provided ‘more information on mode of action for non-genotoxic compounds’. (**31**)

## Discussion and conclusion

Bernstein’s (1955) life-cycle theory of regulatory agencies posits that, when first established in the aftermath of some public disaster, they exhibit regulatory zeal to protect public (health) interests, but then become captured by regulated industries (administrative drift) until a new disaster reinvigorates a regulatory resurgence, commencing a new cycle. Capture theory’s first phase is irrelevant here because the FDA long pre-dated carcinogenicity-testing standards, while the EMA resulted from Europeanization politics, rather drug disasters (Abraham & Lewis, 2000; Carpenter, 2010). Nonetheless, we maintain that developments in carcinogenicity-testing standards since ICH can be understood in terms of the second and third phases, namely, ‘capture’ and ‘regulatory resurgence’, albeit with some theoretical innovations.

Those innovations involve less absolute and dramatic interpretations of ‘capture’ and ‘regulatory resurgence’ than Bernstein. We allow for relativity of capture, ‘boundary-work’ as a facilitator of capture, and ‘reputation’ more broadly defined as a generator of regulatory resurgence (Carpenter, 2010; Jasanoff, 1990). For instance, in arguing that the FDA was captured by industry interests, we do not claim that it was completely so, while also finding that it was less captured than the EMA regarding carcinogenic risk assessment. Going beyond classical capture theory, we appreciate the importance of boundary-work in representing administrative drift towards industry interests as pursuit of techno-scientific progress. Moreover, we found that regulatory resurgence was manifest without a new *public disaster* because regulatory agencies acted to protect their reputations of scientific credibility among the wider expert community when faced with a crisis in regulatory science posing *risks* to the public.

In these terms, capture theory extends to processes concerned with legitimation of risk assessment standards supposed to protect public health when regulatory agencies cede organizational control of those processes to industry. The evidence suggests that capture theory provides a more plausible social scientific understanding of the development of carcinogenicity-testing standards than a theory of regulatory learning from techno-scientific progress. Specifically, the GEM-tests’ validation process did not seek maximum scientific knowledge about their validity, but instead was an exercise, accommodated by regulators, in establishing whether the tests were consistent with industry interests by prioritizing avoidance of false positives. Similarly, our findings suggest that industry framing of GEM-tests’ use around commercial preoccupations with costs and maximization of product success was tolerated by regulators longer than would be expected if regulatory agencies had been merely on a scientific learning curve to improve public-health protective carcinogenic risk assessment.

That resulted in use of GEM tests that rarely recorded positive results for carcinogenicity, together with a marginalization of those more likely to be positive, because of firms’ strategies to avoid commercially damaging positive results. It also reinforced a tendency, already present in the validation process, not to sufficiently interrogate false negativity of GEM tests because negative carcinogenicity results helped drugs advance to the market. Consequently, we suggest that regulators’ acceptance of extensive industrial control of the validation and use of GEM tests obfuscated the impending crisis with the p53 test as an adequate tool of carcinogenic risk assessment to protect public health. The fact that stronger regulatory oversight did not emerge until there was a crisis further implies regulatory resurgence to reassert reputation following capture, rather than a progressive scientific learning curve.

Finally, given experts’ and regulators’ acknowledgement of GEM tests’ limitations in screening for carcinogens, our research raises questions about whether ICH’s pledge that regulations permitting just one lifespan carcinogenicity study in rats plus a GEM test, instead of two-rodent-species lifespan carcinogenicity tests, would not compromise drug safety may not have been upheld. Indeed, our findings raise the spectre that changes to pharmaceutical carcinogenicity-testing standards and subsequent ‘validation’ may have been a massive exercise of boundary-work in which the politico-economic project of decreasing the chance that companies’ products are defined as carcinogenic has masqueraded as a purely ‘techno-scientific’ process.

**Table:** 

Interview notes
1. EU-ICH regulator, pre-clinical drug safety assessment. For discussion of contestations surrounding long-term rodent studies, see Abraham and Reed (2003).
2. US regulator/director, senior FDA adviser.
3. UK university toxicology professor, industry/regulatory consultant.
4. Senior UK regulator.
5. Scientific director, Danish pharmaceutical firm (an ILSI company).
6. Senior FDA representative; National Center for Toxicogenomics (NCT) representative, NIEHS.
7. Professor of pathology & ILSI-ACT committee member.
8. Executive vice-president, US pharmaceutical company & ILSI-ACT member.
9. NCT representative, NIEHS; Vice-president, US pharmaceutical firm (formerly senior FDA scientist); group leader, transgenic carcinogenesis, Laboratory of Molecular Toxicology, NIEHS & ILSI-ACT.
10. Vice-president, safety assessment, Swiss pharmaceutical company; group leader, transgenic carcinogenesis, Laboratory of Molecular Toxicology, NIEHS & ILSI-ACT; government scientist, Netherlands National Institute of Public Health and Environment (NIPHE); NCT representative, NIEHS.
11. NCT representative, NIEHS.
12. Vice-president, safety assessment, Swiss pharmaceutical company.
13. Vice-president, safety assessment, Swiss pharmaceutical company; EU-ICH regulator, pre-clinical drug safety assessment.
14. Toxicologist, vice-president, UK pharmaceutical company.
15. Member, UKCoC.
16. Vice-president, drug safety, US pharmaceutical company & ILSI-ACT; vice-president, safety assessment, Swiss pharmaceutical company; pathologist at US pharmaceutical firm; government scientist, Netherlands NIPHE & ILSI-ACT.
17. FDA-ICH regulator.
18. Pathologist at US pharmaceutical firm.
19. Consultant toxicologist, UK contract research company involved in ILSI-ACT.
20. US regulator; Vice-president, safety assessment, US pharmaceutical company.
21. Distinguished industry toxicologist.
22. Chief Scientist, Laboratory of Experimental Pathology, NIEHS.
23. Senior researcher, carcinogenesis, UK chemical company; vice-president, drug safety, US pharmaceutical firm & ILSI-ACT; toxicologist, UK pharmaceutical company.
24. Associate director, chemical carcinogenesis, NIEHS; government toxicologist/pathologist/geneticist, Netherlands NIPHE.
25. Genetic/cellular toxicologist, US pharmaceutical company & ILSI-ACT.
26. FDA-ICH regulator; vice-president, safety assessment, US pharmaceutical company.
27. Head of US science advisory committee.
28. Senior researcher, carcinogenesis, UK chemical company; vice-president, safety assessment, US pharmaceutical company; executive vice-president, pharmaceutical/safety sciences, US pharmaceutical firm & ILSI-ACT; vice-president, pre-clinical safety, US pharmaceutical company & ICH.
29. Vice-president, drug safety, US pharmaceutical company & ILSI-ACT.
30. Associate director, chemical carcinogenesis, NIEHS.
31. German regulator/genetic toxicologist involved with EMEA and ILSI.

## Figures and Tables

**Table 1:** Interview response rates.

	UK	Other EU	US	Overall (%)
Pharmaceutical industry	4/8	3/10	8/9	15/27 (56%)
Government regulators	3/5	2/9	4/5	9/19 (47%)
Research scientists	5/10	5/8	6/8	16/26 (62%)
Other interests groups	5/9	4/6	4/6	13/21 (62%)
Overall (%)	17/32 (53%)	14/33 (42%)	22/28 (79%)	53/93 (57%)
